# Dermatology in Ghana: a retrospective review of skin disease at the Korle Bu Teaching Hospital Dermatology Clinic

**DOI:** 10.11604/pamj.2017.26.125.10954

**Published:** 2017-03-03

**Authors:** Brooke E Rosenbaum, Rebecca Klein, Paa Gyasi Hagan, Mark-Young Seadey, Naa Larteley Quarcoo, Rachel Hoffmann, Maria Robinson, Margaret Lartey, Marie C Leger

**Affiliations:** 1 New York University School of Medicine, New York, USA; 2 University of Chicago Pritzker School of Medicine, Chicago, USA; 3 Department of Psychiatry, Columbia University College of Physicians and Surgeons, USA; 4 Dermatology Unit, Department of Medicine, Korle Bu Teaching Hospital, Ghana; 5 The Ronald Perelman Department of Dermatology, New York University School of Medicine, USA; 6 Dermatology Unit, Department of Medicine & Therapeutics, University of Ghana School of Medicine and Dentistry, Ghana; 7 Department of Dermatology, Weill Cornell Medical College, New York, USA

**Keywords:** Dermatology, Ghana, atopic dermatitis, scabies

## Abstract

**Introduction:**

Ghana is currently developing its provision of dermatology services. Epidemiologic studies of the skin diseases seen by Ghanaian dermatologists are needed to guide these efforts. We aimed to describe the skin conditions seen by and management practices of Ghanaian dermatologists in a specialized clinic.

**Methods:**

We conducted a chart review of new patients presenting to the Korle Bu Teaching Hospital dermatology clinic during 2014.

**Results:**

Among the 529 patients studied, 700 discrete diagnoses were made. The most commonly diagnosed skin conditions were infections (24.6%) and dermatitis (24.6%); atopic dermatitis (8.4%), acne vulgaris (5.3%) and scabies (5.1%) were the most common specific diagnoses. Among infants, children, and adolescents, the most common diagnosis was atopic dermatitis (31.7%, 30.0%, and 14.9%, respectively). Acne vulgaris (12.0%) was the most common skin condition diagnosed in young adults. Irritant contact dermatitis (6.9%) was most common among adults. Lichen planus (9.9%) was the most commonly diagnosed skin condition in the senior population. Diagnoses made by dermatologists differed from the referral diagnosis documented by primary care providers for 65.8% of patients. The most frequently recommended treatments were antihistamines (47.8%) and topical steroids (38.4%). Only 18 diagnostic biopsies were performed.

**Conclusion:**

Our study summarizes the skin diseases seen and management practices of Ghanaian dermatologists in a specialized clinic at a large public teaching hospital. The results of this study can help to guide future dermatology education and development efforts in Ghana.

## Introduction

Skin disease is a significant cause of morbidity in sub-Saharan Africa [1–5]. Studies conducted in Mali showed that skin disease has an overall prevalence of 34.0% and motivates 11.7% of outpatient primary care visits [1, 6]. Cutaneous infections such as pyoderma and scabies are endemic to African adults and children [4, 5, 7–10]. International public health initiatives have been created to reduce the prevalence of high-morbidity skin diseases such as Buruli ulcers and lymphatic filariasis in Africa [11, 12]. Epidemiological studies of the burden of skin disease in Africa have helped shape directions for physician training as well as public health campaigns. Fewer than 25 certified dermatologists serve the entire population of Ghana, a rapidly developing West African country of approximately 25 million people. To improve access to quality skin care, the Ghana Society of Dermatology, founded in 2011, aims to develop Ghana’s local capacity of dermatologists. In conjunction with the Ghana College of Physicians and Surgeons, they established Ghana’s first and only dermatology training fellowship at Korle Bu Teaching Hospital (KBTH), the largest public hospital in Accra, in 2012. The hospital currently employs 3 full-time dermatologists and 1 part-time dermatologist, as well as 3 trainees in the fellowship program. The KBTH outpatient dermatology clinic operates twice weekly and sees approximately 50 patients per week. Patients generally need a referral from their primary care physician in order to make an appointment at the clinic, although some patients do arrive without a referral and are seen on a case-by-case basis. Empirical data about the local burden of disease can be used to help guide educational and developmental efforts. An understanding of the spectrum of skin disease currently managed by dermatologists in Ghana is needed, as a study of skin conditions at a dermatology clinic in Ghana has not been conducted in over 20 years [13, 14]. Therefore, we conducted a retrospective review of skin conditions seen in the dermatology clinic at KBTH. Our study describes the spectrum of skin disease seen by dermatologists in a specialized dermatology clinic at a large public teaching hospital in Ghana.

## Methods

We performed a retrospective chart review of all new patients presenting to the KBTH dermatology clinic over a 1-year period from January through December 2014. This study was approved by the Institutional Review Boards at NYU Langone Medical Center and Korle Bu Teaching Hospital. All information was obtained from hand-written paper charts used in the dermatology clinic. Several charts that fit criteria for inclusion were unavailable at the time of the study. Charts were reviewed for demographic and clinical information such as sex, age, tribe, home town/city, occupation, and referral diagnosis, date of visit, diagnostic tests, diagnoses, and treatment recommendations. All final diagnoses were made by a dermatologist based on skin examination, laboratory results, and/or biopsy findings. Descriptive statistics were calculated for key demographics, diagnoses, diagnostic tests, and treatment recommendations.

## Results

**Figure 1:**
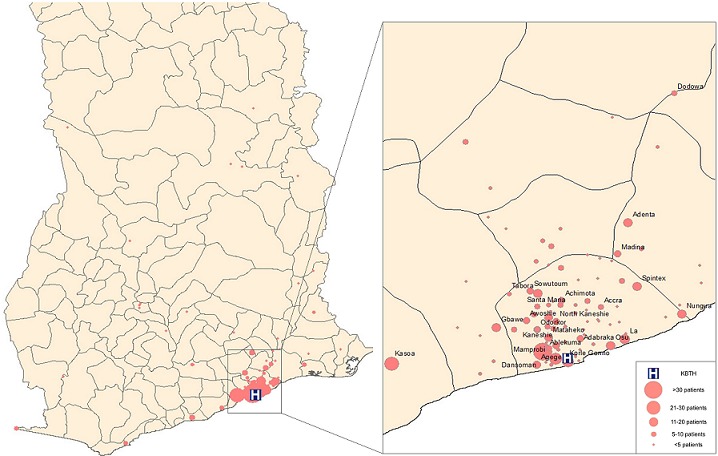
Map of patient-reported hometowns in Ghana

During the study period, 631 new patients were seen in the KBTH dermatology clinic, of which 529 had charts available for review (Table 1). The median age was 28 years old (range 1 month to 105 years old) and 55.7% of patients were female. Most patients belonged to the Ga (23.1%) or Akan (22.9%) tribes, and identified as Christian (92.1%). The most common occupations (≥18 years) were student (22.8%), trader (14.8%), and service industry employee (7.3%). The geographic distribution of patient-reported hometowns is shown in Figure 1. A total of 700 discrete diagnoses were made during the study period (full classification of diseases can be viewed in Online Resource 1). There were 165 patients diagnosed with more than 1 condition. The most commonly seen disease categories were infections (24.6%) and dermatitis/eczema (24.6%). Malignant and pre-malignant conditions were rare (2.8%). Overall, atopic dermatitis (8.4%), acne vulgaris (5.3%), and scabies (5.1%) were the most commonly diagnosed diseases (Table 2). The most common diagnoses among men were atopic dermatitis (7.9%), scabies (5.6%), and warts (5.6%), while atopic dermatitis (8.8%), pityriasis rosea (5.3%), and lichen planus (5.3%) were most common among women (Table 2). Among infants, children, and adolescents, the most common diagnosis was atopic dermatitis (31.7%, 30.0%, and 14.9%, respectively; Table 3). Acne vulgaris (12.0%) was the most common skin condition diagnosed in young adults, irritant contact dermatitis (6.9%) was most common among adults, and lichen planus (9.9%) was the most commonly diagnosed skin condition in the senior population (Table 3). Seventy-one percent of charts contained the referral diagnosis made by the patient's primary care provider. The diagnosis made by dermatologists differed from the original referral diagnosis in 65.8% of these patients. Biopsies were performed on 18 patients, contributing to the following diagnoses: psoriasis (x2), chronic actinic dermatitis, lichen planus, lichen striatus, dermatitis herpetiformis, bullous pemphigoid (x2), sebaceous cyst, vitiligo, mixed connective tissue disease, morphea, granuloma annulare, cutaneous lymphoma, Kaposi sarcoma (x2), and benign neoplasms (x2). Additionally, 98 patients underwent additional laboratory blood testing, 16 had a fungal scraping, and 6 underwent additional radiologic imaging studies. The most frequently recommended treatments were antihistamines (47.8%), topical steroids (38.4%), and keratolytics (27.0%; Online Resource 2). The number of visits per patient ranged from 1 to 21 visits; 40.6% of patients visited the dermatology clinic only once during the study period.

**Table 1:** Patient demographics

Demographic	N (%)
**Sex**	
Male	234 (44.3)
Female	294 (55.7)
**Age group (years)**	
Infant (0-2)	31 (5.9)
Child (3-12)	53 (10.0)
Adolescent (13-18)	32 (6.0)
Young adult (19-30)	159 (30.1)
Adult (31-50)	147 (27.8)
Senior (>50)	107 (20.2)
**Tribe**	
Ga	119 (23.1)
Akan	118 (22.9)
Ewe	107 (20.7)
Fanti	65 (12.6)
Other	107 (20.7)
**Religion**	
Christian	487 (92.1)
Muslim	32 (6.0)
Other	10 (1.9)
**Marital Status (>18 years old)**	
Single	214 (54.0)
Married	171 (43.2)
Divorced	8 (2.0)
Other	3 (0.8)
**Occupation (>18 years old)**	
Student	91 (22.8)
Sales/Trader	59 (14.8)
Retired	30 (7.5)
Service Industry	29 (7.3)
Unemployed	27 (6.8)
Healthcare	24 (6.0)
Finance	21 (5.3)
Education	19 (4.8)
Administrative	16 (4.0)
Business	12 (3.0)
Police/Security	12 (3.0)
Other	60 (15.0)

**Table 2:** Top 10 diagnoses overall and by gender

Overall (*N=700)*	N (%)	Males (*N=302)*	N (%)	Females (*N=396)*	N (%)
Atopic dermatitis	59 (8.4)	Atopic dermatitis	24 (7.9)	Atopic dermatitis	35 (8.8)
Acne vulgaris	37 (5.3)	Scabies	17 (5.6)	Pityriasis rosea	21 (5.3)
Scabies	36 (5.1)	Warts	17 (5.6)	Lichen planus	21 (5.3)
Irritant contact dermatitis	33 (4.7)	Acne vulgaris	16 (5.3)	Acne vulgaris	20 (5.1)
Lichen planus	26 (3.7)	Irritant contact dermatitis	14 (4.6)	Scabies	19 (4.8)
Seborrhoeic dermatitis	25 (3.6)	Seborrhoeic dermatitis	11 (3.6)	Irritant contact dermatitis	19 (4.8)
Warts	23 (3.3)	Tinea pedis	10 (3.3)	Vitiligo	14 (3.5)
Vitiligo	22 (3.1)	Pityriasis versicolor	9 (3.0)	Papular urticaria	13 (3.3)
Pityriasis versicolor	17 (2.4)	Chronic urticaria	9 (3.0)	Seborrhoeic dermatitis	13 (3.3)
Pityriasis rosea	16 (2.3)	Vitiligo	8 (2.6)	Allergic contact dermatitis	9 (2.3)
				Acquired ichthyosis	9 (2.3)

**Table 3:** Top 5 diagnoses distributed by age group

Infants (0-2)(*N=41)*	N (%)	Children (3-12) (*N=70)*	N (%)	Adolescents (13-18) (*N=47)*	N (%)
Atopic dermatitis	13 (31.7)	Atopic dermatitis	21 (30.0)	Atopic dermatitis	7 (14.9)
Impetigo	3 (7.3)	Papular urticaria	4 (5.7)	Scabies	4 (8.5)
Scabies	3 (7.3)	Molluscum	3 (4.3)	Pruritic papular dermatitis	2 (4.3)
Contact dermatitis	2 (4.9)	Pruritic papular dermatitis	3 (4.3)	Papular urticaria	2 (4.3)
Epidermolysis bullosa	2 (4.9)	Scabies	3 (4.3)	Exfoliative dermatitis	2 (4.3)
Papular urticaria	2 (4.9)	Tinea capitis	3 (4.3)	Acne vulgaris	2 (4.3)
Vitiligo	2 (4.9)	Vitiligo	3 (4.3)		
**Young Adults (19-30)** *N=209*	**N (%)**	**Adults (31-50) (***N=188)*	**N (%)**	**Seniors (>50) (***N=141)*	**N (%)**
Acne vulgaris	25 (12.0)	Irritant contact dermatitis	13 (6.9)	Lichen planus	14 (9.9)
Scabies	14 (6.7)	Warts	11 (5.9)	Tinea pedis	6 (4.3)
Seborrheic dermatitis	13 (6.2)	Acne vulgaris	9 (4.8)	Irritant contact dermatitis	6 (4.3)
Pityriasis versicolor	12 (5.7)	Scabies	8 (4.3)	Vitiligo	6 (4.3)
Irritant contact dermatitis	11 (5.3)	Seborrhoeic dermatitis	8 (4.3)	Exfoliative dermatitis	5 (3.5)

## Discussion

Until recent years, Ghana lacked a sustainable workforce of dermatologists and had limited access to resources necessary to providing quality skin care. In order to facilitate Ghana’s recent efforts to improve its local dermatologic capacity, we described the spectrum of skin disease seen in a specialized dermatology clinic at the largest public teaching hospital in Accra. Our results provide an updated overview of the main skin diseases managed by Ghanaian dermatologists, and may be used to guide future dermatology education efforts and public health campaigns. Additionally, our data can help to train primary care physicians in recognizing and treating the most common dermatologic conditions, as they currently manage the majority of skin complaints in Ghana. There have been few prior studies of skin diseases observed in specialty dermatology clinics in Ghana. One study by Addo et al. surveyed skin disease seen in the KBTH dermatology clinic between 1987-1988 [13]. The study reported dermatitis and eczema as the causes of 44.8% of patient visits, while infectious etiologies accounted for 16.8% [13]. However, another study conducted by Doe et al. in 1995 at the Komfo Anokye Teaching Hospital dermatology clinic in Kumasi found more skin disease associated with infections (46.3%) than eczematous rashes (18.4%) [14]. While no studies of pediatric skin disease seen in specialty clinics are reported in the literature, there have been a few studies conducted of the prevalence of specific skin conditions in Ghanaian children-including acne, tinea capitis, and eczema [4, 15–17]. We found atopic dermatitis to be the most commonly diagnosed condition at the KBTH clinic. While our observation of atopic dermatitis was similar to that of Addo et al. (16.5%), the previous study found contact dermatitis to be much more common (27.6%) [13]. Doe et al. found dermatitis to be the second most common diagnosis (18.4%), but this was mostly due to irritant contact dermatitis and seborrheic dermatitis [14]. Interestingly, 2 studies conducted in specialty dermatology clinics in Nigeria reported a much lower incidence of atopic dermatitis (6% and 3%, respectively) [18, 19].

Acne vulgaris comprised 5.3% of skin diagnoses reported in our study. Similarly, Doe et al. found that acne vulgaris accounted for 4.6% of visits [14]. While Addo et al. found acne vulgaris to be relatively uncommon (2%), the author speculated that the actual incidence of acne vulgaris was higher than that reported because Ghanaians perceive acne as a normal feature of adolescence and rarely seek medical treatment [13]. The increased incidence of acne observed in our study may reflect changing attitudes towards the condition. The high frequency of infections reported in our study is consistent with Doe et al. in which 46.3% of diagnoses were of infectious etiology [14]. Similarly Doe et al. also found scabies (12.4%) to be the most common cause of infectious skin disease. Scabies can significantly affect quality of life as it can cause severe pruritus and untreated scabies can result in bacterial superinfection. Overcrowding is an important driver in the spread of scabies, although the associations of hygiene and socioeconomic status with scabies infestation are debated, since scabies is present in developed countries of all socioeconomic classes [1, 20]. Our study found a low incidence of malignant and pre-malignant skin diseases. This result is consistent with Doe et al., which found that malignant and pre-malignant tumors accounted for only 0.5% of diagnoses [14]. Of the malignant tumors seen in our study, 6 were Kaposi sarcoma and 3 were cutaneous T-cell lymphoma, but no basal cell carcinomas (BCC), squamous cell carcinomas (SCC) or malignant melanomas (MM) were observed. Similarly, in Addo et al., only 1 SCC was reported [13]. However, studies in Nigeria and Mali reported a larger range of skin malignancies including BCC, SCC, and MM [3, 21]. The discrepancy in the incidence of skin malignancies may be due to the relatively low prevalence of albinism in Ghana compared to that of Nigeria and Mali, or to differences in biopsy capabilities and access to pathologists among the West African countries [22].

Our results reflect important issues in the provision of dermatologic care in Ghana in general, and at KBTH more specifically. Skin biopsies are performed relatively infrequently in Ghana, due primarily to logistical constraints and lack of dermatopathologists. Clinical diagnoses are favored, while biopsies are reserved for cases where there is significant diagnostic uncertainty or when the condition does not respond to empirical therapy. Another important logistical consideration in Ghana is that payment for medical services-both the physician visit and any ancillary laboratory testing-must be made at the time medical care is received. Similarly, medications are purchased out-of-pocket and the available formulary is limited; inexpensive and readily-available formulations are therefore preferred [23]. Finally, at KBTH connective tissue disorders are primarily treated by rheumatologists rather than dermatologists, leading to the misleadingly low prevalence of these disorders in our data. Our study demonstrates that Ghanaian dermatologists provide extremely valuable expertise, as approximately 65% of diagnoses made by the dermatologists at KBTH differed from the referring diagnoses listed by primary care physicians. The high rate of diagnosis change also suggests a potential opportunity for education at the primary care level. Indeed, results of similar epidemiological studies in sub-Saharan Africa have been used to create a management algorithm for common skin diseases for use by local primary care physicians [24]. Similarly, the low number of biopsies observed during the study also highlights an opportunity for additional education in dermatopathology. There were several limitations of this study to consider. First, the source of our data was patient records of a specialized dermatology clinic at a tertiary care hospital. This type of study carries the inherent selection bias of patients that actively sought out specialty care for their conditions, and does not represent the incidence of skin disease throughout the country. Additionally, KBTH is one of few hospitals in Ghana with multiple practicing dermatologists and a Department of Dermatology, as well as the only hospital with a dermatology fellowship training program. Therefore, our may not be generalizable to all dermatology clinics in Ghana, especially in rural areas where resources may be scarcer.

## Conclusion

Twenty-five years ago, dermatology in Ghana was considered to be “a hobby of just any physician” [13]. The creation of the first dermatology training fellowship at KBTH represents a significant step towards attaining sustainable and accessible dermatologic care for all Ghanaians. Our study summarizes the skin diseases commonly seen by Ghanaian dermatologists and can help guide future dermatology education efforts in Ghana. Future population-based studies are needed to further characterize the extent of skin disease affecting the country.

### What is known about this topic

- According to the most recent study of skin diseases seen by dermatologists in Ghana (Addo 1990), the most common conditions managed by dermatologists were dermatitis/eczema (44.8%) and infections (16.8%).

### What this study adds

- An updated understanding the local burden of disease managed by dermatologists is necessary to guide the ongoing dermatology education and development efforts in Ghana, including the country's first dermatology fellowship program;
- We describe the skin conditions seen, management practices, and needs of Ghanaian dermatologists in a specialized clinic at a large public teaching hospital;
- The results of this study can help to guide the ongoing dermatology education and development efforts in Ghana.
